# Redetermination of the crystal structure of K_2_Hg(SCN)_4_

**DOI:** 10.1107/S2056989017009148

**Published:** 2017-06-27

**Authors:** Jascha Bandemehr, Matthias Conrad, Florian Kraus

**Affiliations:** a Anorganische Chemie, Fachbereich Chemie, Philipps-Universität Marburg, Hans-Meerwein-Strasse 4, 35032 Marburg

**Keywords:** crystal structure, redetermination, mercury, thio­cyanate

## Abstract

Single crystals of K_2_Hg(SCN)_4_ [dipotassium tetra­thio­cyanato­mercurate(II)] were grown from aqueous solutions of potassium thio­cyanate and mercury(II) thio­cyanate and studied by single-crystal X-ray diffraction. In comparison with the previously reported structure model [Zvonkova (1952 ▸). *Zh. Fiz. Khim*. **26**, 1798–1803], all atoms in the crystal structure were located, with lattice parameters and fractional coordinates determined to a much higher precision. In the (crystal) structure, the Hg^II^ atom is located on a twofold rotation axis and is coordinated in the form of a distorted tetra­hedron by four S atoms of the thio­cyanate anions. The K^+^ cation shows a coordination number of eight.

## Abstract 2

The redetermination of the crystal structure of potassium tetra­thio­cyanato­mercurate(II) reveals all atoms tombe located and shows much higher precision and accuracy in comparison with the previously determined structure.

## Chemical context   

In search for suitable educts for fluorination we thought that K_2_Hg(SCN)_4_ would be a well-suited candidate. Once we had obtained the compound, we noticed that the original structure determination (Zvonkova, 1952 ▸) was of low precision with the light atoms (C and N) not determined, so we redetermined the crystal structure to much higher precision and accuracy.

K_2_Hg(SCN)_4_ was first synthesized in 1901 (Rosenheim & Cohn, 1901 ▸) by adding an aqueous solution of potassium thio­cyanate to a boiling solution of mercury(II) thio­cyanate and crystallization upon cooling to room temperature. The crystal structure has been known since 1952 (Zvonkova, 1952 ▸) and IR spectra were first measured in 1962 (Tramer, 1962 ▸). Related compounds of the type *A*_2_Hg(SCN)_4_ with *A* = Rb, Cs, NH_4_, NMe_4_ are also known (Larbot & Beauchamp, 1973 ▸; Tramer, 1962 ▸). The Hg^II^ atom in K_2_Hg(SCN)_4_ is coordinated in the form of a distorted tetra­hedron by four S atoms in a fashion similar to the Hg^II^ atom in the structure of CoHg(SCN)_4_ (Jefferey & Rose, 1968 ▸). Such tetra­hedrally coordinated Hg^II^ atoms are also known, for example, for the halide and pseudo-halide compounds *A*_2_Hg*X*_4_, *viz*. Cs_2_HgBr_4_ (Pakhomov *et al.*, 1978 ▸; Altermatt *et al.*, 1984 ▸; Pinheiro *et al.*, 1998 ▸), Cs_2_HgCl_4_ (Linde *et al.*, 1983 ▸; Pakhomov *et al.* 1992*a* ▸,*b* ▸; Bagautdinov & Brown, 2000 ▸), Cs_2_HgI_4_ (Zandbergen *et al.*, 1979 ▸; Pakhomov & Fedorov, 1973 ▸), K_2_Hg(CN)_4_ (Gerlach & Powell, 1986 ▸; Dickinson, 1922 ▸) and Rb_2_Hg(CN)_4_ (Klüfers *et al.*, 1981 ▸).

## Structural commentary   

**Figure 1:**
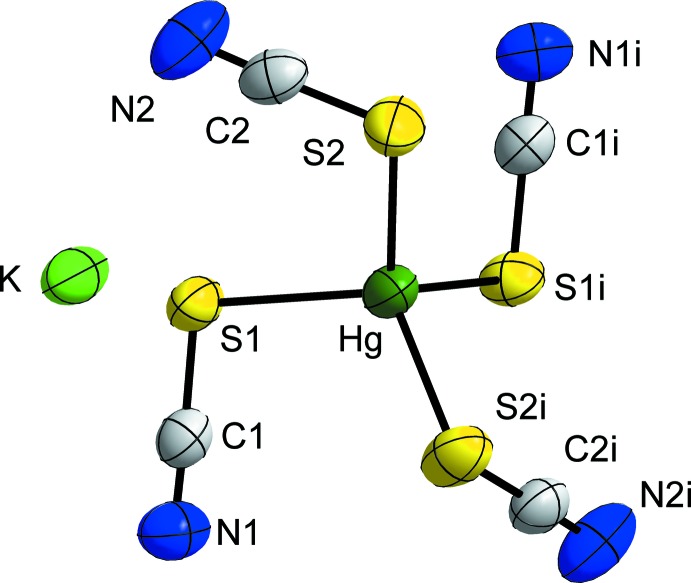
A section of the crystal structure of K_2_Hg(SCN)_4_, showing the [Hg(SCN)_4_]^2−^ anion and the K^+^ cation. Displacement ellipsoids are shown at the 70% probability level at 293 K. [Symmetry code: (i) −*x*, *y*, 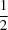 − *z*].

The lattice parameters obtained by our room-temperature single-crystal structure determination (Table 1 ▸) agree with those obtained previously (*a* = 11.04, *b* = 9.22, *c* = 13.18 Å, *β* = 106.30°, *Z* = 4; Zvonkova, 1952 ▸). K_2_Hg(SCN)_4_ crystallizes in the monoclinic crystal system in space group *C*2/*c* (No. 15). The Hg^II^ atom is located on a twofold rotation axis (Wyckoff position 4*e*) and is coordinated in the form of a distorted tetra­hedron by four S atoms of the thio­cyanate anions (Fig. 1 ▸). The S—Hg—S angles are in the range 105.02 (2)–114.67 (3)° and the Hg—S distances are 2.5380 (8) and 2.5550 (7) Å, both in good agreement with the previously reported data (S—Hg—S angle: 102–118°, Hg—S distance: 2.54 (2); Zvonkova, 1952 ▸). The Hg—S distance is slightly longer than those of the sixfold-coordinated Hg^II^ atom in Hg(SCN)_2_ [2.381 (6) Å] (Beauchamp & Goutier, 1972 ▸) and lies within the range of Hg—S distances [2.3954 (11)–2.7653 (6) Å] for the threefold coordinated Hg^II^ atom in KHg(SCN)_3_ (Weil & Häusler, 2014 ▸).

As may be expected, the two unique SCN^−^ anions are almost linear [178.0 (3), 178.2 (3)°], and the angles are comparable with those reported for Hg(SCN)_2_ [177.5 (13)°; Beauchamp & Goutier, 1972 ▸] or KHg(SCN)_3_ [176.41 (4)–179.8 (3)°; Weil & Häusler, 2014 ▸]. The S—C [1.656 (3), 1.665 (3) Å] and C—N [1.153 (5), 1.152 (4) Å] distances are comparable as well [S—C: 1.62 (2), C—N: 1.18 (3) Å] (Beauchamp & Goutier, 1972 ▸) [S—C: 1.657 (4)–1.675 (3) Å, C—N: 1.140 (4)–1.145 (5) Å] (Weil & Häusler, 2014 ▸). The Hg—S—C angles in the title salt are 98.59 (10) and 97.06 (10)°, respectively. In comparison with the coordination polyhedron of the Hg^II^ atom and the structural feature of the SCN^−^ anions in CoHg(SCN)_4_ [Hg—S: 2.558–2.614 Å, S—C: 1.635–1.720 Å, C—N: 1.200–1.322 Å, S—Hg—S angles: 105.1 (1), 108.7 (1)°, Hg—S—C angle: 97.3 (5)°] (Jefferey & Rose, 1968 ▸), the respective angles and distances of the complex [Hg(SCN)_4_]^2−^ anion presented here agree well. In total, a [Hg(SCN)_4_]^2−^ anion is surrounded by twelve potassium atoms.

**Figure 2:**
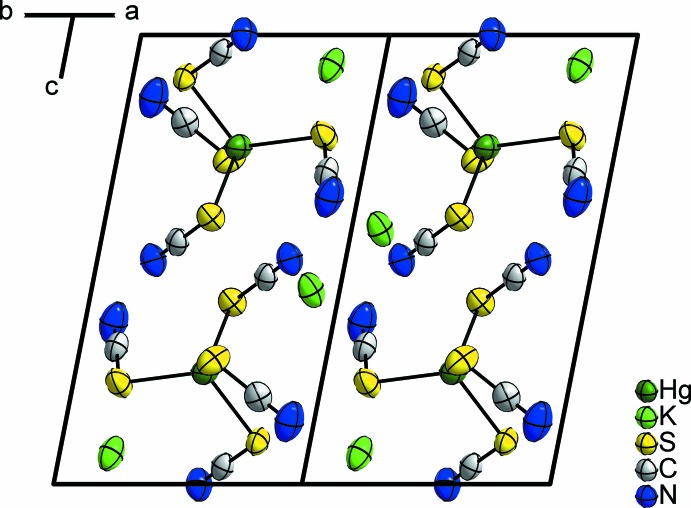
The crystal structure of K_2_Hg(SCN)_4_ viewed along [110]. Displacement ellipsoids are shown at the 70% probability level at 293 K. Bonds involving the K^+^ cation are omitted for clarity.

The K^+^ cation shows a coordination number of eight, with disparate bond lengths that can be associated with a [4 + 3 + 1] coordination. Four K—N distances are in the range 2.816 (4)–3.031 (5) Å, three K—S distances are in the range 3.4466 (11)–3.5315 (12) Å and there is one very long K—N distance of 3.793 (5) Å. Therefore, the resulting coordination polyhedron is of an odd shape. The K^+^ cation is coordinated in total by five [Hg(SCN)_4_]^2−^ units, three of these in a monodentate manner (two *via* N atoms and one *via* the S atom of the thio­cyanate anions) and the other two in a bidentate mode (*via* the N and S atoms of neighboring thio­cyanate anions). Overall, a complex three-dimensional framework results. The crystal structure of the title compound is shown in Fig. 2 ▸.

## Synthesis and crystallization   

Potassium tetra­thio­cyanato­mercurate(II) was synthesized by slowly adding a potassium thio­cyanate solution (2.076 g, 21.36 mmol in 10 ml H_2_O) to a boiling solution of mercury(II) thio­cyanate (3.176 g, 10.03 mmol in 10 ml H_2_O). After the formed mercury sulfide had been filtered off through a Büchner funnel, the solution was concentrated on a hot plate until crystallization set in. The crystallized product was collected on a Büchner funnel and the filtrate was allowed to stand at room temperature until crystals of much better quality were obtained. A selected colorless single crystal was investigated by X-ray diffraction. Mercury(II) thio­cyanate was prepared as reported previously (Hermes, 1866 ▸) using mercury(II) nitrate and potasium thio­cyanate and was recrystallized out of ethanol.

## Refinement   

Crystal data, data collection and structure refinement details are summarized in Table 1 ▸. As a starting model for the structure refinement, the atomic coordinates of the previously reported K_2_Hg(SCN)_4_ structure model were used (Zvonkova, 1952 ▸). The positions of the C and N atoms were located from a difference-Fourier map.

## Supplementary Material

Crystal structure: contains datablock(s) I. DOI: 10.1107/S2056989017009148/wm5399sup1.cif

Structure factors: contains datablock(s) I. DOI: 10.1107/S2056989017009148/wm5399Isup2.hkl

CCDC reference: 1556957

Additional supporting information:  crystallographic information; 3D view; checkCIF report

## Figures and Tables

**Table 1:** Experimental details

Crystal data	
Chemical formula	K_2_Hg(SCN)_4_
*M*_r_	511.11
Crystal system, space group	Monoclinic, *C*2/*c*
Temperature (K)	293
*a*, *b*, *c* (Å)	10.8154 (9), 9.3243 (7), 13.3313 (11)
β (°)	106.648 (6)
*V* (Å^3^)	1288.05 (18)
*Z*	4
Radiation type	Mo *K*α
μ (mm^−1^)	13.21
Crystal size (mm)	0.24 × 0.15 × 0.12

Data collection	
Diffractometer	Stoe *IPDS* 2T
Absorption correction	Integration (*X-RED32* and *X-SHAPE*; Stoe & Cie, 2009 ▸)
*T*_min_, *T*_max_	0.103, 0.344
No. of measured, independent and observed [*I* > 2σ(*I*)] reflections	14009, 2710, 2298
*R*_int_	0.043
(sin θ/λ)_max_ (Å^−1^)	0.798

Refinement	
*R*[*F* ^2^ > 2σ(*F* ^2^)], *wR*(*F* ^2^), *S*	0.024, 0.053, 1.08
No. of reflections	2710
No. of parameters	70
Δρ_max_, Δρ_min_ (e Å^−3^)	1.15, −0.75

Computer programs: *X-AREA* (Stoe & Cie, 2011 ▸), *X-RED* (Stoe & Cie, 2009 ▸), *SHELXT2014* (Sheldrick, 2015*a* ▸), *SHELXL2014* (Sheldrick, 2015*b* ▸), *DIAMOND* (Brandenburg, 2015 ▸) and *publCIF* (Westrip, 2010 ▸).

## supplementary crystallographic information

### Crystal data

**Table:**

K_2_Hg(SCN)_4_	*F*(000) = 936
*M**_r_* = 511.11	*D*_x_ = 2.636 Mg m^−^^3^
Monoclinic, *C*2/*c*	Mo *K*α radiation, λ = 0.71073 Å
*a* = 10.8154 (9) Å	Cell parameters from 25154 reflections
*b* = 9.3243 (7) Å	θ = 2.9–35.0°
*c* = 13.3313 (11) Å	µ = 13.21 mm^−^^1^
β = 106.648 (6)°	*T* = 293 K
*V* = 1288.05 (18) Å^3^	Block, colourless
*Z* = 4	0.24 × 0.15 × 0.12 mm

### Data collection

**Table:**

Stoe IPDS 2T diffractometer	2710 independent reflections
Radiation source: sealed X-ray tube, 12 x 0.4 mm long-fine focus	2298 reflections with *I* > 2σ(*I*)
Plane graphite monochromator	*R*_int_ = 0.043
Detector resolution: 6.67 pixels mm^-1^	θ_max_ = 34.6°, θ_min_ = 2.9°
rotation method scans	*h* = −17→17
Absorption correction: integration (X-RED32 and X-SHAPE; Stoe & Cie, 2009)	*k* = −14→14
*T*_min_ = 0.103, *T*_max_ = 0.344	*l* = −21→21
14009 measured reflections	

### Refinement

**Table:**

Refinement on *F*^2^	Primary atom site location: other
Least-squares matrix: full	Secondary atom site location: other
*R*[*F*^2^ > 2σ(*F*^2^)] = 0.024	*w* = 1/[σ^2^(*F*_o_^2^) + (0.022*P*)^2^ + 1.7*P*] where *P* = (*F*_o_^2^ + 2*F*_c_^2^)/3
*wR*(*F*^2^) = 0.053	(Δ/σ)_max_ = 0.001
*S* = 1.08	Δρ_max_ = 1.15 e Å^−^^3^
2710 reflections	Δρ_min_ = −0.75 e Å^−^^3^
70 parameters	Extinction correction: SHELXL2014 (Sheldrick, 2015b), Fc^*^=kFc[1+0.001xFc^2^λ^3^/sin(2θ)]^-1/4^
0 restraints	Extinction coefficient: 0.0086 (2)

### Special details

**Table:**

Geometry. All esds (except the esd in the dihedral angle between two l.s. planes) are estimated using the full covariance matrix. The cell esds are taken into account individually in the estimation of esds in distances, angles and torsion angles; correlations between esds in cell parameters are only used when they are defined by crystal symmetry. An approximate (isotropic) treatment of cell esds is used for estimating esds involving l.s. planes.

### Fractional atomic coordinates and isotropic or equivalent isotropic displacement parameters (Å^2^)

**Table:**

	*x*	*y*	*z*	*U*_iso_*/*U*_eq_	
Hg	0.0000	0.52099 (2)	0.2500	0.03803 (7)	
K	0.16185 (7)	1.04695 (8)	0.43195 (7)	0.04817 (17)	
S1	0.10639 (7)	0.68302 (8)	0.40531 (6)	0.03720 (14)	
S2	0.18135 (7)	0.36527 (10)	0.22498 (7)	0.04701 (18)	
C1	−0.0130 (3)	0.6793 (3)	0.4611 (2)	0.0332 (5)	
C2	0.3046 (3)	0.4505 (3)	0.3055 (3)	0.0387 (6)	
N1	−0.0940 (3)	0.6801 (3)	0.5013 (3)	0.0462 (6)	
N2	0.3926 (3)	0.5077 (4)	0.3607 (4)	0.0615 (9)	

### Atomic displacement parameters (Å^2^)

**Table:**

	*U*^11^	*U*^22^	*U*^33^	*U*^12^	*U*^13^	*U*^23^
Hg	0.02612 (7)	0.05275 (11)	0.03471 (8)	0.000	0.00789 (5)	0.000
K	0.0336 (3)	0.0491 (4)	0.0637 (4)	−0.0053 (3)	0.0169 (3)	−0.0164 (3)
S1	0.0294 (3)	0.0399 (3)	0.0426 (3)	−0.0077 (2)	0.0107 (2)	−0.0070 (3)
S2	0.0350 (3)	0.0567 (5)	0.0486 (4)	0.0053 (3)	0.0108 (3)	−0.0162 (3)
C1	0.0307 (11)	0.0271 (11)	0.0399 (12)	−0.0003 (9)	0.0070 (9)	−0.0026 (9)
C2	0.0304 (12)	0.0385 (15)	0.0475 (14)	0.0066 (10)	0.0115 (10)	0.0066 (11)
N1	0.0408 (13)	0.0437 (14)	0.0586 (17)	−0.0011 (11)	0.0215 (12)	−0.0039 (12)
N2	0.0346 (14)	0.0530 (19)	0.087 (3)	0.0011 (12)	0.0023 (14)	0.0011 (16)

### Geometric parameters (Å, º)

**Table:**

Hg—S2	2.5380 (8)	K—K^iv^	4.4669 (15)
Hg—S2^i^	2.5380 (8)	S1—C1	1.665 (3)
Hg—S1^i^	2.5550 (7)	S1—K^v^	3.5316 (12)
Hg—S1	2.5551 (7)	S2—C2	1.656 (3)
K—N2^ii^	2.816 (4)	S2—K^viii^	3.4865 (12)
K—N1^iii^	2.823 (3)	C1—N1	1.152 (4)
K—N1^iv^	2.860 (3)	C1—K^iv^	3.529 (3)
K—N2^v^	3.031 (5)	C2—N2	1.153 (5)
K—C2^vi^	3.408 (3)	C2—K^viii^	3.408 (3)
K—C2^v^	3.414 (3)	C2—K^v^	3.414 (3)
K—S1	3.4466 (11)	N1—K^ix^	2.823 (3)
K—S2^vi^	3.4865 (12)	N1—K^iv^	2.860 (3)
K—S1^v^	3.5315 (12)	N2—K^x^	2.816 (4)
K—C1^iv^	3.529 (3)	N2—K^v^	3.031 (5)
K—K^vii^	4.3913 (14)		
			
S2—Hg—S2^i^	110.21 (4)	S1—K—C1^iv^	131.89 (5)
S2—Hg—S1^i^	114.67 (3)	S2^vi^—K—C1^iv^	161.89 (6)
S2^i^—Hg—S1^i^	105.02 (2)	S1^v^—K—C1^iv^	121.11 (5)
S2—Hg—S1	105.02 (2)	N2^ii^—K—K^vii^	122.43 (8)
S2^i^—Hg—S1	114.67 (3)	N1^iii^—K—K^vii^	39.71 (6)
S1^i^—Hg—S1	107.50 (4)	N1^iv^—K—K^vii^	39.09 (6)
N2^ii^—K—N1^iii^	161.33 (10)	N2^v^—K—K^vii^	86.73 (7)
N2^ii^—K—N1^iv^	83.58 (10)	C2^vi^—K—K^vii^	117.52 (6)
N1^iii^—K—N1^iv^	78.80 (9)	C2^v^—K—K^vii^	70.39 (6)
N2^ii^—K—N2^v^	80.42 (13)	S1—K—K^vii^	159.02 (4)
N1^iii^—K—N2^v^	100.55 (9)	S2^vi^—K—K^vii^	116.01 (3)
N1^iv^—K—N2^v^	74.44 (9)	S1^v^—K—K^vii^	97.01 (3)
N2^ii^—K—C2^vi^	91.55 (12)	C1^iv^—K—K^vii^	53.40 (5)
N1^iii^—K—C2^vi^	94.70 (8)	N2^ii^—K—K^iv^	41.99 (10)
N1^iv^—K—C2^vi^	128.94 (9)	N1^iii^—K—K^iv^	136.17 (7)
N2^v^—K—C2^vi^	154.57 (9)	N1^iv^—K—K^iv^	75.38 (6)
N2^ii^—K—C2^v^	98.21 (12)	N2^v^—K—K^iv^	38.43 (7)
N1^iii^—K—C2^v^	81.16 (8)	C2^vi^—K—K^iv^	129.03 (5)
N1^iv^—K—C2^v^	68.68 (8)	C2^v^—K—K^iv^	56.66 (5)
N2^v^—K—C2^v^	19.47 (8)	S1—K—K^iv^	73.46 (2)
C2^vi^—K—C2^v^	161.02 (7)	S2^vi^—K—K^iv^	136.14 (3)
N2^ii^—K—S1	72.82 (7)	S1^v^—K—K^iv^	97.61 (3)
N1^iii^—K—S1	125.84 (7)	C1^iv^—K—K^iv^	58.43 (5)
N1^iv^—K—S1	148.83 (6)	K^vii^—K—K^iv^	107.42 (3)
N2^v^—K—S1	81.66 (7)	C1—S1—Hg	97.06 (10)
C2^vi^—K—S1	72.91 (5)	C1—S1—K	96.25 (9)
C2^v^—K—S1	94.40 (6)	Hg—S1—K	133.66 (3)
N2^ii^—K—S2^vi^	111.59 (10)	C1—S1—K^v^	102.56 (10)
N1^iii^—K—S2^vi^	80.81 (6)	Hg—S1—K^v^	102.38 (3)
N1^iv^—K—S2^vi^	146.21 (6)	K—S1—K^v^	117.55 (2)
N2^v^—K—S2^vi^	136.13 (7)	C2—S2—Hg	98.59 (10)
C2^vi^—K—S2^vi^	27.76 (5)	C2—S2—K^viii^	73.50 (11)
C2^v^—K—S2^vi^	133.75 (5)	Hg—S2—K^viii^	109.15 (3)
S1—K—S2^vi^	63.89 (2)	N1—C1—S1	178.0 (3)
N2^ii^—K—S1^v^	127.71 (8)	N1—C1—K^iv^	46.43 (18)
N1^iii^—K—S1^v^	68.47 (7)	S1—C1—K^iv^	131.85 (12)
N1^iv^—K—S1^v^	123.33 (7)	N2—C2—S2	178.2 (3)
N2^v^—K—S1^v^	68.09 (7)	N2—C2—K^viii^	100.5 (3)
C2^vi^—K—S1^v^	99.48 (6)	S2—C2—K^viii^	78.74 (12)
C2^v^—K—S1^v^	61.72 (5)	N2—C2—K^v^	61.1 (3)
S1—K—S1^v^	62.45 (2)	S2—C2—K^v^	119.82 (14)
S2^vi^—K—S1^v^	72.06 (2)	K^viii^—C2—K^v^	160.38 (10)
N2^ii^—K—C1^iv^	71.53 (9)	C1—N1—K^ix^	127.6 (2)
N1^iii^—K—C1^iv^	92.39 (7)	C1—N1—K^iv^	116.6 (2)
N1^iv^—K—C1^iv^	16.96 (7)	K^ix^—N1—K^iv^	101.20 (9)
N2^v^—K—C1^iv^	61.48 (8)	C2—N2—K^x^	149.3 (3)
C2^vi^—K—C1^iv^	138.43 (8)	C2—N2—K^v^	99.4 (3)
C2^v^—K—C1^iv^	60.50 (7)	K^x^—N2—K^v^	99.58 (13)

Symmetry codes: (i) −*x*, *y*, −*z*+1/2; (ii) *x*−1/2, *y*+1/2, *z*; (iii) *x*+1/2, *y*+1/2, *z*; (iv) −*x*, −*y*+2, −*z*+1; (v) −*x*+1/2, −*y*+3/2, −*z*+1; (vi) −*x*+1/2, *y*+1/2, −*z*+1/2; (vii) −*x*+1/2, −*y*+5/2, −*z*+1; (viii) −*x*+1/2, *y*−1/2, −*z*+1/2; (ix) *x*−1/2, *y*−1/2, *z*; (x) *x*+1/2, *y*−1/2, *z*.
